# Agonism at mGluR2 receptors reduces dysfunctional checking on a rodent analogue of compulsive-like checking in obsessive compulsive disorder

**DOI:** 10.1007/s00213-025-06774-2

**Published:** 2025-04-03

**Authors:** Colin McKenzie, Bart Sloot, Felippe Espinelli Amorim, Trevor W Robbins, Amy L Milton

**Affiliations:** 1 Department of Psychology, University of Cambridge, Downing Site, Cambridge, CB2 3EB UK; 2 University of Radboud, Nijmegen, The Netherlands

**Keywords:** Obsessive-compulsive disorder, Metabotropic glutamate receptor, Rat, Checking, Observing response task

## Abstract

**Rationale:**

Obsessive-compulsive disorder (OCD) affects 1–3% of the population. Current therapies, including selective serotonin reuptake inhibitors, are not universally effective in managing OCD. Recent discoveries indicating hyperactivation of key regions within the corticostriatal thalamic circuitry that supports OCD, and alterations in the ratio of glutamate: GABA in regions such as the anterior cingulate cortex, suggest that drugs targeting glutamatergic signalling may be effective in reducing OCD symptoms.

**Objectives:**

This study sought to determine whether two drugs targeting metabotropic glutamate receptors could reduce excessive checking behaviour in a rodent analogue of compulsive-like checking in OCD, the Observing Response Task (ORT).

**Methods:**

Rats were trained on the ORT and separately classified on a pavlovian autoshaping task to identify the subpopulation of sign-trackers, which show higher levels of excessive checking. Once responding had stabilised, rats received systemic administration of different doses of the mGluR2 positive allosteric modulator AZD-8529 and its vehicle in a Latin square design, and the effects on ORT performance were assessed. Following completion of AZD-8529 dosing, a subset of rats received administration of different doses of the mGluR2/3 agonist LY404039 and its vehicle in a Latin square design, and ORT performance assessed.

**Results:**

Both AZD-8529 and LY404039 produced dose-dependent reductions in checking behaviour, including at doses that did not impair generalised measures of task performance.

**Conclusions:**

The similarity in effect of AZD-8529 and LY404039 suggests that the capacity of these drugs to reduce checking is mediated by mGluR2s, which may provide a promising target for future treatment development for OCD.

**Supplementary Information:**

The online version contains supplementary material available at 10.1007/s00213-025-06774-2.

## Introduction

Obsessive-compulsive disorder (OCD) is a severe and disabling condition estimated to affect up to 3% of the population, and costing the UK National Health Service in excess of £3.7bn annually (Kochar et al. 2023). OCD is characterised by intrusive obsessional thoughts and the performance of compulsions, of which multiple subtypes have been identified (Mataix-Cols et al. 2005). Common symptom dimensions include contamination fears and excessive handwashing, hoarding, order and symmetry, and excessive checking (Olatunji et al. 2007; Radomsky and Rachman 2004; Wahl et al. 2008). Current treatments range from the behavioural (exposure with response prevention), to the pharmacological, including medication with selective serotonin reuptake inhibitors (SSRIs) and dopamine D_2_ receptor antagonists. For severe, treatment-refractory OCD, neurosurgical and neuromodulatory interventions, including deep brain stimulation, may be warranted (Fineberg et al. 2018; Robbins et al. 2019). However, the efficacy of these treatments is somewhat limited, and current psychological and pharmacotherapies remain ineffective for a significant proportion of OCD patients (Kochar et al. 2023). Thus, there is a clear need for the development of novel pharmaceutical approaches (Pittenger 2015).

One pharmaceutical target of increasing interest in the treatment of OCD is the glutamatergic system (Pittenger et al. 2011). At the circuit level, OCD is associated with dysfunctional corticostriatal thalamic circuitry, particularly increased metabolic activity in regions including the striatum, anterior thalamus, orbitofrontal cortex and the anterior cingulate cortex (Maia et al. 2008), which may suggest hyperactivation and glutamatergic dysfunction. OCD patients have been found to have elevated levels of glutamate in cerebrospinal fluid (Bhattacharyya et al. 2009), and recent studies assessing the ratio of glutamate and GABA expression in the anterior cingulate cortex using magnetic resonance spectroscopy have indicated a shift in the ratio, towards increased glutamatergic transmission and hyperexcitability in patients with OCD (Biria et al. 2024). Thus, a reduction in glutamatergic signalling might be predicted to ameliorate this hyperactivity and thereby reduce OCD symptoms.

While there has been great interest in the use of the NMDA receptor antagonist ketamine to treat treatment-refractory OCD, following on from the discovery of its rapid anti-depressant effects (Zarate et al. 2006) and promising results from relatively small-scale clinical trials (Bloch et al. 2012; Rodriguez et al. 2011, 2013), the difficulties associated with ketamine administration (Rodriguez et al. 2017) and its status as a controlled substance in many countries indicates the value of exploring alternative glutamatergic targets. One such target that has received surprisingly little attention is the modulation of presynaptic metabotropic glutamate receptors (mGluRs), of which the class 2 and 3 receptors mediate the inhibition of glutamate release (Schoepp 2001). Although an mGluR2/3 agonist pomaglumetad (LY404039) has been trialled for the treatment of schizophrenia (Kinon et al. 2015), no such drug has been tested for its impact on OCD symptoms.

Testing of new treatment strategies relies upon the use of translational animal models, and one such behavioural animal model with relevance to OCD is the Observing Response Task (Eagle et al. 2014). This task produces excessive and unnecessary checking behaviour in a subpopulation of animals trained on a volatile, two-lever task. Briefly, rats are trained to work for sucrose rewards by pressing levers, but due to the volatility of both the reinforcement schedule and the location of the correct lever (which changes in an unsignalled manner throughout the session), rats are unable to predict with certainty which lever will provide reward. However, they can gain information about the current contingencies by making an ‘observing response’ on a ‘checking’ lever at the rear of the chamber. This briefly illuminates a cue above the currently correct lever, and the majority of animals use this information adaptively to guide their subsequent responding. However, a subpopulation of rats, which are particularly sensitive to pavlovian influences on the behaviour (‘sign-trackers’; Eagle et al. 2020; Vousden et al. 2020) show excessive interest and pressing of the checking lever. These ‘extra observing lever presses’ have no programmed consequences, are not associated with reward delivery, or even extension of the light cue that highlights the currently correct lever. These dysfunctional responses are considered reflective of the excessive checking observed in OCD patients, supported by the finding that when tested on the human version of the Observing Response Task (Morein-Zamir et al. 2018), OCD patients show increased certainty seeking (Morein-Zamir et al. 2016).

Thus, this study sought to investigate in rats the impact of using two different drugs targeting mGluR2s; the mGluR2 positive allosteric modulator AZD-8529 and the mGluR2/3 agonist LY404039 (pomaglumetad) on levels of checking on the Observing Response Task.

## Materials and methods

### Subjects

Subjects were 96 behaviourally naïve male Lister-Hooded rats (Charles River, UK) weighing 212–310 g at the start of the experiments. All rats were housed in groups of 2–4 rats per cage in a humidity- and temperature-controlled (21^o^C) vivarium under a reversed light-dark cycle (lights on at 1900 h, lights off at 0700 h), with a cardboard tube as enrichment. Rats were allowed to acclimatise to the animal facility for 7 days prior to the start of any experimental procedures. During this time, they had *ad libitum* access to food and water. Prior to the start of behavioural procedures, the animals were food-restricted such that they were maintained at 90–95% of their age-matched free-feeding weight, being fed after the completion of each day’s behavioural procedures. This was in addition to any food earned during the behavioural procedures. This research was regulated under the UK Animals (Scientific Procedures) Act 1986 Amendment Regulations (2012) on Project Licence PA9FBFA9F following ethical review by the University of Cambridge Animal Welfare and Ethical Review Body.

### Apparatus

The rats were trained in twelve operant conditioning chambers (29.5 × 32.5 × 23.5 cm; Med Associates, St. Albans, VT, USA) situated in sound-attenuating boxes. Each chamber was equipped with two 4-cm wide retractable levers 8 cm above the grid floor and 12 cm apart. Above each lever was a white cue light (2.5 W, 24 V), and a white houselight (2.5 W, 24 V) was at the top of the opposite wall and illuminated throughout the behavioural sessions. The levers flanked a food receptacle where 45 mg sucrose pellets (TestDiet, Opcobe, UK) were delivered. Illumination of the lights above the levers referred to the lever being active and thus giving a food reward. At the back of the chamber a third, observing, lever was present. In sessions where the observing lever was extended, pressing the observing lever illuminated the light above the active lever if it was previously unlit. Chamber operation and on-line data collection were controlled with the Observing Response Task program (written by A.C. Mar) on the Whisker Server platform (Cardinal and Aitken 2010).

### Behavioural procedures

**Fig. 1:**
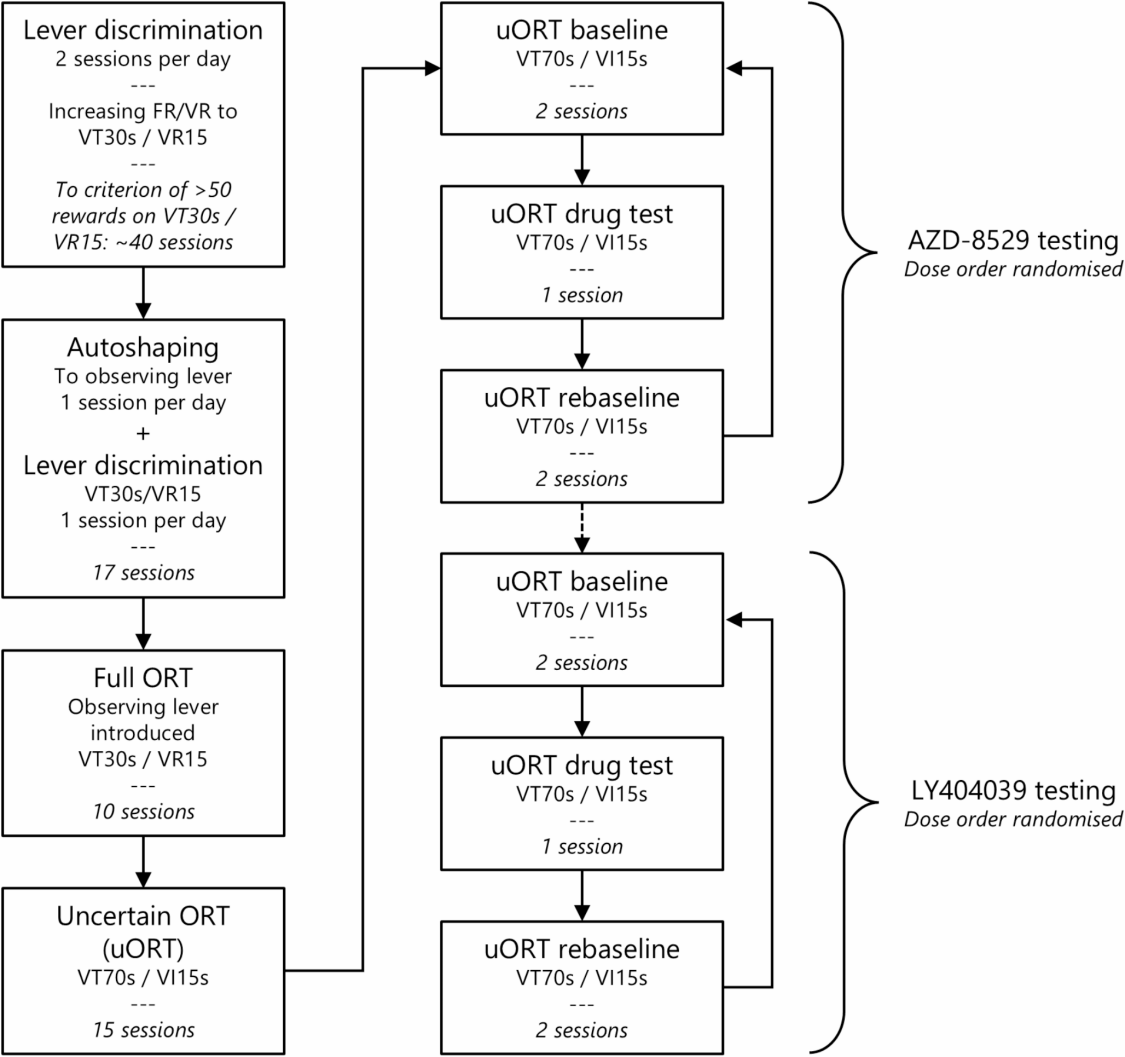
Overview of behavioural procedures. Rats were initially trained on lever discrimination, during which an active lever was reinforced on a progressively leaner schedule and the active and inactive levers switched on a variable time (VT) 30s schedule (range: 15–45 s). Lever discrimination training progressed to a criterion of earning more than 50 reinforcers per session, with the active lever reinforced on a variable ratio (VR) 15 schedule (range: 10–20). Training progressed to include autoshaping and lever discrimination training for 17 sessions before rats progressed to the full ORT, in which the active lever was reinforced on a VR15 schedule and the levers switched on a VT30s schedule. Pressing of the observing lever led to illumination of a cue light above the active lever for 15 s on the first press of a bout only, with subsequent presses being classified as extra observing lever presses (eOLPs). After 10 sessions of the full ORT, rats underwent the ‘uncertain ORT’ (uORT), where active lever pressing was reinforced on a variable interval (VI) 15s schedule (range: 10–20 s) and the active and inactive levers switched on a VT70s schedule (range: 20–120 s). Following 15 sessions of uORT, rats progressed to drug testing, beginning with AZD-8529 and, for some rats (indicated by dashed line), followed by LY404039 (see text for details). All drug testing involved two baseline uORT sessions, a single drug test uORT session with the order of doses counterbalanced in a Latin Square design, and two rebaseline uORT sessions. This pattern continued until drug testing was completed

Rats were trained on the Observing Response Task (ORT) as described previously (Vousden et al. 2020). Briefly, rats were trained on the ORT and underwent separate autoshaping training sessions to enable classification into goal-tracking and sign-tracking phenotypes. Animals were subsequently tested in the ORT under conditions of uncertainty (uORT), where it was more difficult for animals to discriminate between the active and inactive levers. Following three weeks of training on the uORT, rats then underwent drug testing over several weeks, using an incomplete block design in which rats received different doses of drugs in a counterbalanced order. For the first two days of each week, rats were run on the uORT to establish their pre-drug baseline levels of responding. On the third day, they received a dose of drug prior to the uORT session. On the fourth and fifth days, they underwent uORT sessions to determine any long-lasting effects of the drug manipulation, and to rebaseline their responding. Figure 1 provides an overview of behavioural procedures.

### Observing response task

Animals were trained on the ORT as previously described (Vousden et al. 2020). Rats were initially trained to discriminate between active and inactive levers, with the identity of the levers changing during the sessions on a variable time (VT) 30s schedule (range: 15–45 s) schedule during initial training. The identity of the active lever was signalled by illumination of the cue light located above the lever. The observing lever remained retracted during these initial sessions. Responses on the active lever were reinforced initially on a fixed ratio (FR) 1 schedule by the presentation of a sucrose pellet (45 mg TestDiet sucrose pellets, Opcobe, UK) in the food magazine, before advancing through progressively leaner FR and variable ratio (VR) schedules based on the animals’ performance in each session. Ultimately, all rats progressed to responding on a VR15 (range: 10–20) schedule. These initial behavioural training sessions were conducted twice a day with a break of approximately 45 min between sessions. When discrimination training was entering its final stages, rats underwent autoshaping sessions (see below) for the first session of each day, followed by discrimination training for the second session of each day.

Following reliable discrimination of the active and inactive levers, and completion of the 17 sessions of autoshaping, rats were trained on both the full and ‘uncertainty’ version of the ORT (uORT). For both of these, the observing lever was now presented. Responses on the observing lever illuminated the cue light above the currently active lever for 30 s for the first four sessions of full ORT training, and for 15 s thereafter (and during the uORT). During the full ORT, active lever presses continued to be reinforced on the VR15 schedule (range: 10–20) with the identity of the active lever changing on a VT30s schedule (range: 15–45 s). Following the tenth session of full ORT, rats progressed onto the uORT. This was the same as the full ORT, except that the active lever was reinforced on a variable interval (VI) schedule of 15 s (range: 10–20 s) and the identity of the active lever changed on an VT70s (range: 20–120 s) schedule.

Responses on the ORT were collected automatically by a computer running the Whisker Control server (Cardinal and Aitken 2010). Seven dependent variables were measured during the ORT session: (i) observing lever presses (OLPs), which were functional observing responses that led to illumination of the light above the active lever; (ii) extra observing lever presses (eOLPs), which were responses on the observing lever performed while the cue light was already illuminated. These responses did not prolong the cue light illumination, and therefore these responses had no function; (iii) active lever presses (rate per minute); (iv) inactive lever presses (rate per minute); (v) discrimination (light on); (vi) discrimination (light off) and (vii) rewards earned. The discrimination measures quantified the accuracy of lever pressing with the light on and off, respectively. These were calculated as the number of active lever presses (with the light on or off) divided by total (active and inactive) lever presses (with the light on or off). These values were multiplied by 100 to give accuracy as a percentage, with 50% corresponding to responding at chance.

### Autoshaping

During autoshaping sessions, the lever that would subsequently be used as the observing lever was presented 30 times, in the absence of the two levers on the front of the chamber. Ten seconds after presentation of the observing lever it retracted, which was followed by the delivery of a single sucrose pellet (45 mg TestDiet sucrose pellets, Opcobe, UK) into the food magazine on the other side of the chamber. Rats received a total of 17 autoshaping sessions.

Rats were classified as goal-trackers, sign-trackers or intermediates based on their behaviour during these sessions, by an experimenter who remained blind to their ORT performance. Classification occurred in a similar fashion to that described in Vousden et al. (2020), where the number of lever approaches and the number of nosepokes made during the CS were used to calculate a ratio of conditioned stimulus: magazine approaches. This ratio was averaged across the final two sessions of autoshaping, and the distribution of ratios plotted. Clear subpopulations were observed in the distributions, with group differences observed in CS approaches and magazine approaches (Supplementary Figs. 1 and 2) and the data were cross-checked with previous classifications using this method to ensure consistency across cohorts. Animals classified as intermediates (*n* = 15) were excluded from drug dosing and subsequent analyses.

### Drugs

Rats received systemic injections of AZD-8529 (Med Chem Express), a potent positive allosteric modulator of the class 2 subtype of metabotropic glutamate receptor (mGluR2) and LY404039 (Merck Life Science, UK), an agonist at mGluR2/3 receptors. AZD-8529 was administered intraperitoneally 2 h prior to behavioural testing in a Latin Square design at doses of 0.3 mg/kg, 1 mg/kg, 3 mg/kg and 10 mg/kg, with rats receiving 2–3 of these doses in addition to a saline vehicle injection (1 ml/kg), in an incomplete block design. 13 rats received a dose of 30 mg/kg, which has been used previously (Justinova et al. 2015). However, due to unexpected adverse effects on the animals (impairment of gut motility and blockage of the gut), these animals were culled and planned 30 mg/kg dosing discontinued. The data for these animals was excluded from all analyses, giving a total *n* = 68 rats included in the analysis for the AZD-8529.

Following AZD-8529 dosing, *n =* 37 rats were given a washout period of at least one week before dosing with LY404039. LY404039 was administered subcutaneously 30 min prior to behavioural testing at doses of 0.3 mg/kg and 1 mg/kg, in addition to a 1 ml/kg water double distilled vehicle injection, in a Latin Square design. These doses were chosen as they have previously been shown to reduce alcohol-seeking behaviour (Rodd et al. 2006). A subset of these animals (*n* = 5) also received an LY404039 dose of 3 mg/kg, but as early analysis of the data revealed generalised impairments of this dose on task performance, this dose was discontinued. Due to the low numbers of animals tested, these data are not presented.

### Statistical analysis

Data are presented as means ± standard error of mean (s.e.m.). Autoshaping data (CS approaches and nosepokes) were analysed using a mixed model analysis of variance (ANOVA), with Session as a within-subjects factor and Phenotype (ST vs. GT) as a between-subjects factor. Due to high individual variability in the ORT data, with differing variances between groups indicated by Levene’s test, lever pressing measures (observing lever presses, extra observing lever presses, active lever presses and inactive lever presses) on the ORT were square-root transformed prior to analysis. Performance at each dose of drug was compared to the average performance of the two sessions prior to drug dosing (‘Baseline’) and to the two sessions following drug dosing (‘Rebaseline’) to determine the persistence of any observed drug effects. The data were analysed using a mixed model ANOVA with Session (Baseline vs. Dose vs. Rebaseline) as a within-subjects factor and Phenotype (ST vs. GT) as a between-subjects factor for each dose of AZD-8529 and LY404039 tested. The confirmation of significant main effects and differences among individual means were further analysed using Šidák-corrected planned comparisons wherever appropriate. The significance level was set at *p* ≤ .05 and for all significant analyses, the effect size was reported by the partial eta-squared value (η^2^). All statistical analyses were performed using SPSS (v.28, IBM).

### Data availability

Data are available at 10.17863/CAM.111643.

## Results

### The mGluR2 positive allosteric modulator AZD-8529 dose-dependently reduced checking behaviour

Both functional Observing Lever Presses (OLPs) and dysfunctional Extra Observing Lever Presses (eOLPs) varied markedly across rats, so the data were square root transformed, and responding under drug was compared to responding during the two sessions prior to dosing (‘Baseline, BL’) and the two sessions following dosing (‘Rebaseline, RBL’).

**Fig. 2:**
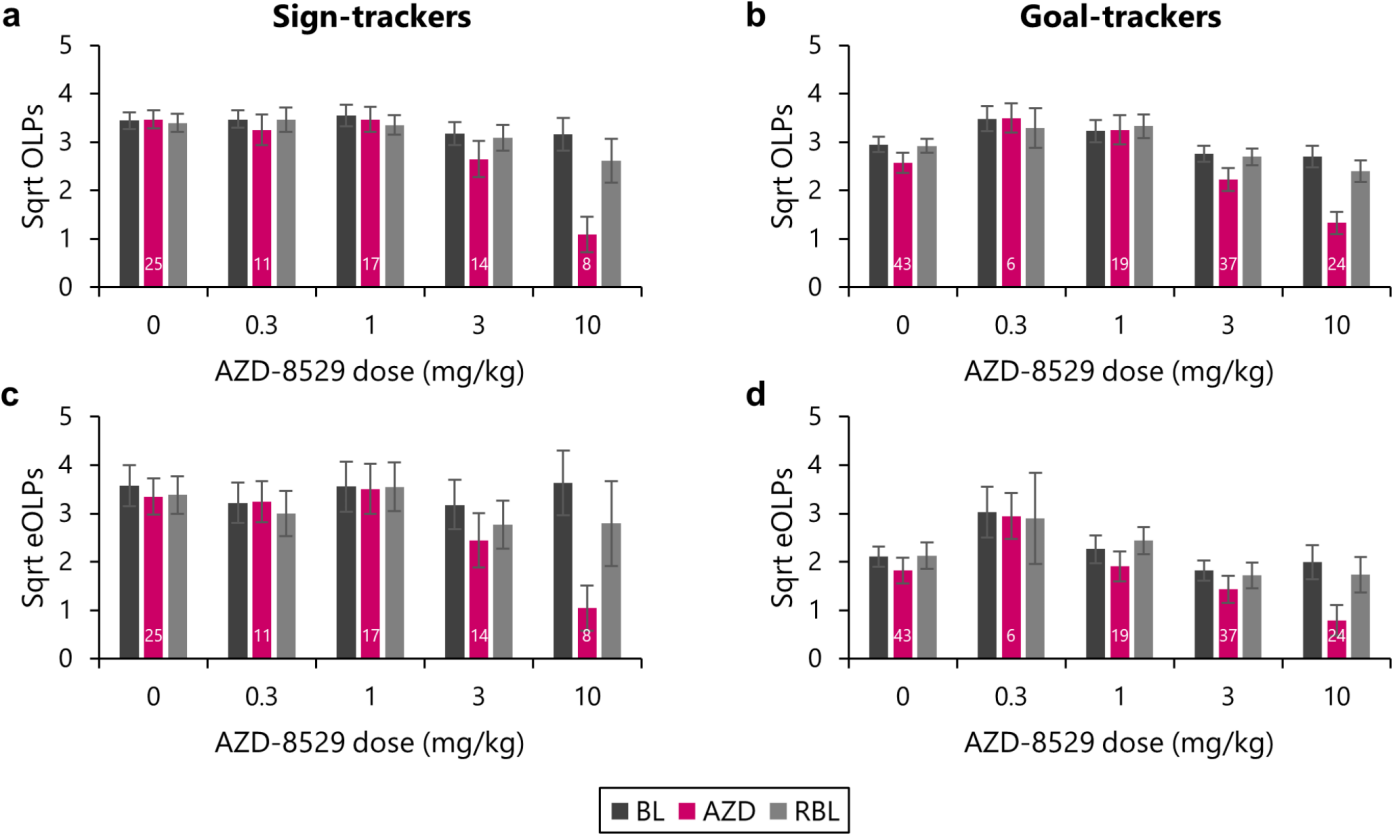
The mGluR2 positive allosteric modulator AZD-8529 dose-dependently reduced functional and dysfunctional checking. Rats were classified as sign-trackers and goal-trackers and trained on the Observing Response Task prior to testing with acute doses of the mGluR2 positive allosteric modulator AZD-8529 in a Latin square design. All rats received treatment with vehicle (0 mg/kg), and a subset of the different AZD-8529 doses. The number of rats receiving each dose is represented by numbers at the base of the bar for the drug dosing day; the same rats are represented in the ‘baseline’ and ‘rebaseline’ bars for each dose. In rats classified as sign-trackers, the 3 mg/kg and 10 mg/kg doses of AZD-8529 reduced **(a)** functional Observing Lever Presses (OLPs) and **(c)** dysfunctional extra Observing Lever Presses (eOLPs). A similar effect was observed in goal-trackers, for both **(b)** functional OLPs and **(d)** dysfunctional eOLPs. BL, baseline sessions; AZD: treatment with AZD-8529 treatment; RBL, rebaseline sessions. Data are shown as means ± s.e.m.

As expected, when rats were dosed with saline vehicle, there were no differences in responding under drug for either OLPs [Figure 2a, b; Session: *F*_(1.8,31.1)_ = 2.40, *p* = .10] or eOLPs [Figure 2c, d; Session: *F*_(2,132)_ = 1.68, *p* = .19]. As we have observed previously, rats classified as sign-trackers showed higher levels of checking, both for OLPs [Phenotype: *F*_(1,66)_ = 6.37, *p* = .014, η^2^ = 0.09; Session x Phenotype: *F*_(1.81,119.5)_ = 3.83, *p* = .028, η^2^ = 0.06] and eOLPs [Phenotype: *F*_(1,66)_ = 11.8, *p* = .001, η^2^ = 0.15]. A similar lack of effect on checking was found for the 0.3 mg/kg dose [OLPs, Session: *F* < 1; eOLPs, Session: *F* < 1] and the 1 mg/kg dose [OLPs, Session: *F* < 1; eOLPs, Session: *F* < 1].

The 3 mg/kg dose of AZD-8529 reduced checking behaviour compared to baseline, for both OLPs [Session: *F*_(1.76,86.0)_ = 8.96, *p* < .001, η^2^ = 0.15] and eOLPs [Session: *F*_(1.87,91.5)_ = 5.46, *p* = .007, η^2^ = 0.10]. Although sign-trackers checked more overall, the 3 mg/kg dose of AZD-8529 was effective at reducing dysfunctional checking in both sign-trackers and goal-trackers [eOLPs, Phenotype: *F*_(1,49)_ = 5.68, *p* = .021, η^2^ = 0.10; Session x Phenotype: *F* < 1]. As we observed previously, sign-trackers and goal-trackers showed similar levels of functional checking [OLPs, Phenotype: *F*_(1,49)_ = 1.56, *p* = .22] and AZD-8529 reduced functional checking similarly in goal-trackers and sign-trackers [OLPs, Session x Phenotype: *F* < 1].

The 10 mg/kg dose of AZD-8529 also reduced checking behaviour compared to baseline, for OLPs [Session: *F*_(1.83,54.9)_ = 37.0, *p* < .001, η^2^ = 0.55] and eOLPs [Session: *F*_(1.85,55.5)_ = 28.1, *p* < .001, η^2^ = 0.48]. At this dose, dysfunctional checking was affected more in sign-trackers than goal-trackers [eOLPs, Session x Phenotype: *F*_(1.85,55.5)_ = 3.62, *p* = .037, η^2^ = 0.11], with Sidak-corrected pairwise comparisons showing that while sign-trackers showed higher levels of dysfunctional checking during the baseline sessions [*p* = .029], their dysfunctional checking was normalised to the same level as goal-trackers while receiving the 10 mg/kg dose [*p* = .69] and during the post-drug sessions [*p* = .20].

Thus, the mGluR2 positive allosteric modulator AZD-8529 reduced both functional and dysfunctional checking behaviour at the 3 mg/kg and 10 mg/kg doses.

### The 3 mg/kg dose of AZD-8529, which reduced checking, left generalised measures of task performance intact

#### Discrimination between the active and inactive levers, and overall rates of lever pressing

**Fig. 3:**
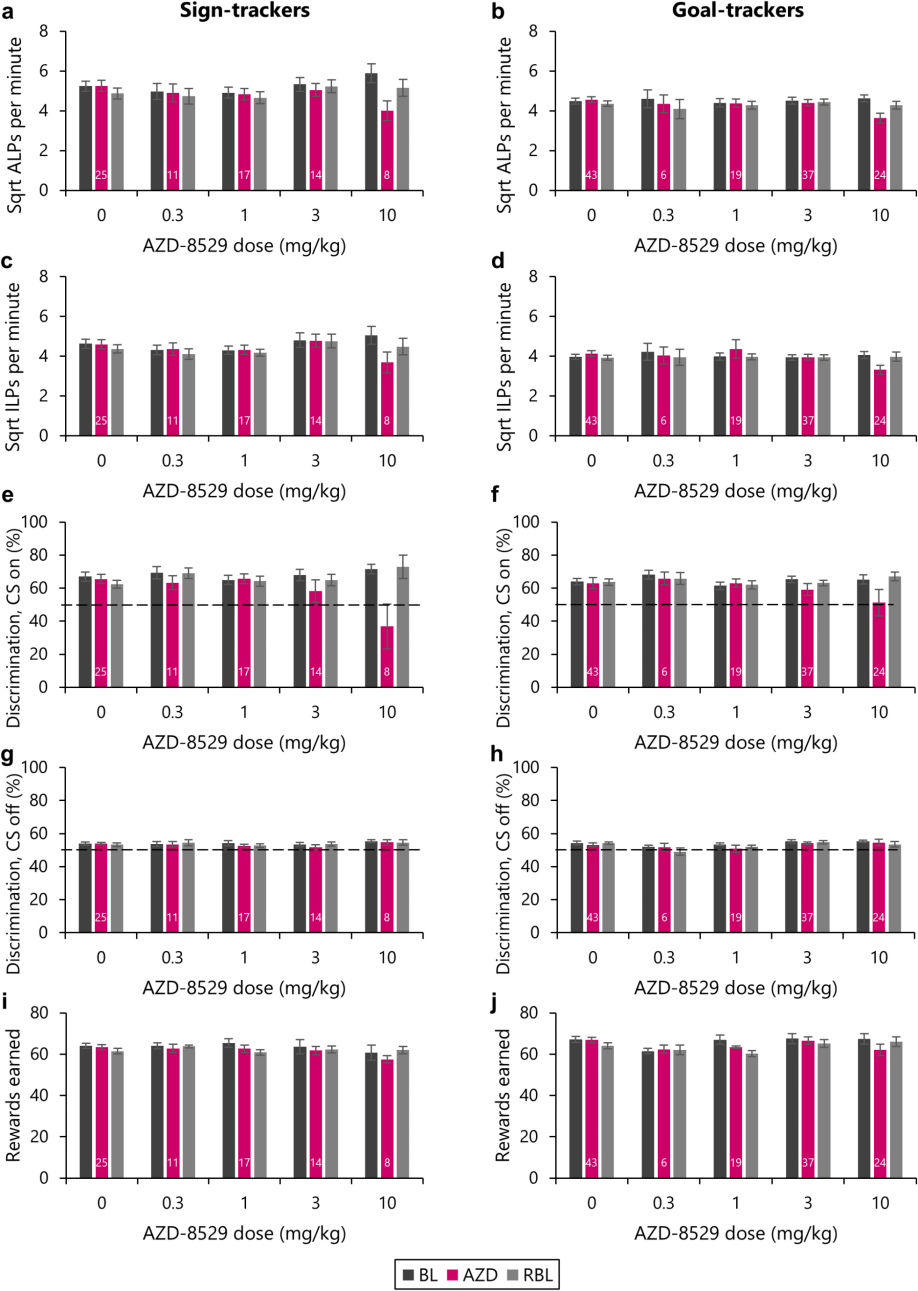
The reductions in checking produced by AZD-8529 were selective at the 3 mg/kg dose but not the 10 mg/kg dose. The effects of AZD-8529 on generalised measures of task performance were assessed. For both sign-trackers **(a**,** c**,** e**,** g**,** i)** and goal-trackers **(b**,** d**,** f**,** h**,** j)** the 10 mg/kg, but not the 3 mg/kg, dose of AZD-8529 reduced rates of **(a**,** b)** active lever pressing and **(b**,** d)** inactive lever pressing, and **(e**,** f)** discrimination between the two levers with the cue light on. For both sign-trackers and goal-trackers, **(g**,** h)** discrimination with the cue light off remained at chance. **(i**,** j)** The 10 mg/kg dose of AZD-8529 also produced a small reduction in the number of rewards earned. Group sizes are shown by the numbers at the base of each bar for the drug dosing day; the same rats are represented in the ‘baseline’ and ‘rebaseline’ bars for each dose. The dotted lines in (e), (f), (g) and (h) represent responding at chance (50%). Data are shown as means ± s.e.m.

To assess the impact of AZD-8529 on task performance more generally, several generalised measures of task performance were compared for each of the doses of AZD-8529 administered. As would be expected for the vehicle-treated condition, rats showed higher rates of lever pressing on the active over the inactive lever across the baseline, drug testing and rebaseline sessions [Figure 3a-d; Lever: *F*_(1,66)_ = 68.4, *p* <.001, η^2^ = 0.51]. Rats tended to respond less during the rebaseline session, but this was the case for both levers [Session: *F*_(2,132)_ = 17.4, *p* <.001, η^2^ = 0.21; Lever x Session: *F* < 1]. Sign-trackers tended to respond more than goal-trackers [Phenotype: *F*_(1,66)_ = 5.66, *p* =.02, η^2^ = 0.08]. Similar effects were observed at the 0.3 mg/kg dose, with rats discriminating between the active and inactive levers, and responding less during the rebaseline sessions [Lever: *F*_(1,15)_ = 10.8, *p* =.005, η^2^ = 0.42; Session: *F*_(1.62,24.3)_ = 6.77, *p* =.007, η^2^ = 0.31; Lever x Session: *F* < 1]. Rats also discriminated between the active and inactive levers at the 1 mg/kg dose of AZD-8529 [Lever: *F*_(1,34)_ = 16.2, *p* <.001, η^2^ = 0.32]. Thus, for the doses of AZD-8529 that did not reduce checking behaviour, lever discrimination remained unimpaired.

Of the two doses of AZD-8529 that reduced checking behaviour, only the 3 mg/kg dose left overall rates of lever pressing intact. Rats continued to discriminate between the active and inactive levers on the 3 mg/kg dose [Lever: *F*_(1,49)_ = 39.1, *p* <.001, η^2^ = 0.44], with no overall change in lever pressing across the baseline, drug treatment and rebaselining sessions [Session: *F* < 1]. The rate of active lever pressing reduced slightly under drug treatment [Lever x Session: *F*_(2,98)_ = 3.95, *p* =.022, η^2^ = 0.08]. Sidak-corrected pairwise comparisons revealed that rats continued to discriminate between the active and inactive levers across the baseline, drug dosing and rebaseline sessions [active vs. inactive lever, all *p*’s < 0.001] but that active lever pressing reduced slightly during the 3 mg/kg dosing session compared to the baseline session [*p* =.023], though not the rebaseline session [*p* =.41]. By contrast, treatment with the 10 mg/kg dose of AZD-8529 reduced rates of lever pressing compared to the baseline and rebaseline sessions. While rats continued to discriminate between the active and inactive levers [Lever: *F*_(1,30)_ = 30.5, *p* <.001, η^2^ = 0.50], responding was reduced during the drug treatment session [Session: *F*_(2,60)_ = 30.5, *p* <.001, η^2^ = 0.50; Sidak-corrected pairwise comparisons showed reduced responding during the drug treatment session compared to the baseline and rebaselining sessions, all *p*’s < 0.001].

Thus, while the 10 mg/kg dose of AZD-8529 reduced checking, it also reduced overall rates of lever pressing. By contrast, the 3 mg/kg dose of AZD-8529 reduced checking without impairing ongoing responding on the active and inactive levers.

#### Discrimination with the cue light on and off

The doses of AZD-8529 that did not affect checking behaviour also did not impair the ability of rats to respond on the correct lever in the presence of the cue light. Under vehicle treatment, rats more readily discriminated between the active and inactive levers when the light cue was illuminated [Figure 3e-h; Cue: *F*_(1,66)_ = 60.2, *p* <.001, η^2^ = 0.48], with greater accuracy in the presence of the light cue being observed in baseline, drug treatment and rebaseline sessions [Session: *F* < 1; Cue x Session: *F* < 1]. This was also the case for the 0.3 mg/kg dose [Cue: *F*_(1,15)_ = 45.5, *p* <.001, η^2^ = 0.75; Session: *F* < 1; Cue x Session: *F* < 1] and the 1 mg/kg dose of AZD-8529 [Cue: *F*_(1,34)_ = 64.3, *p* <.001; η^2^ = 0.65; Session: *F* < 1; Cue x Session: *F*_(1.69,57.6)_ = 1.02, *p* =.36].

The 3 mg/kg dose of AZD-8529, which reduced checking behaviour but left the rate of lever pressing unimpaired, left overall cue-guided responding intact [Cue: *F*_(1,49)_ = 21.2, *p* <.001, η^2^ = 0.30]. Responding was slightly reduced in the drug treatment session relative to the baseline session, but not the rebaseline session [Session: *F*_(1.43,70.0)_ = 4.79, *p* =.02, η^2^ = 0.09; pairwise comparisons showed a reduction in discrimination between the baseline and drug treatment sessions (*p* =.024) but not the drug treatment and rebaseline sessions (*p* =.23)]. Importantly, rats treated with the 3 mg/kg dose of AZD-8529 continued to show better discrimination in the presence of the cue [Cue x Session: *F*_(1.33,65.1)_ = 2.24, *p* =.13]. This was not the case for the 10 mg/kg dose of AZD-8529, which markedly impaired discrimination between the levers when the cue was on [Cue: *F*_(1,30)_ = 3.08, *p* =.09; Session: *F*_(1.25,37.6)_ = 9.00, *p* =.003, η^2^ = 0.23; Cue x Session: *F*_(1.23,36.8)_ = 9.42, *p* =.002, η^2^ = 0.24].

Thus, consistent with the effects on lever pressing, the 10 mg/kg dose of AZD-8529 produced generalised deficits on task performance in addition to reducing checking behaviour, while the 3 mg/kg dose did not.

#### Rewards earned

The number of rewards (Fig. 3i, j) earned on task was generally consistent across individuals, although there were some reductions in the number of rewards earned across the course of a week of testing, i.e. between baselining and rebaselining sessions. However, this did not appear attributable to drug treatment, as this effect was also seen following injections of vehicle [Session: *F*_(1.71,113)_ = 8.33, *p* <.001, η^2^ = 0.11; pairwise comparisons showed that responding was lower in the rebaselining sessions compared to both the baseline and vehicle treatment sessions (*p*’s < 0.007) which, importantly, did not differ from each other (*p* =.87)]. A similar effect was observed for the 1 mg/kg dose of AZD-8529 [Session: *F*_(1.94,65.9)_ = 9.66, *p* <.001, η^2^ = 0.22], though for this dose, pairwise comparisons revealed that the rewards earned during the rebaselining sessions were lower than the baseline (*p* =.001), but that these did not differ from the number of rewards earned under drug treatment (*p* =.088), which in turn did not differ from the rewards earned during the baseline sessions (*p* =.059). There were no differences in the number of rewards earned under the 0.3 mg/kg dose of AZD-8529 [Session: *F* < 1].

The 3 mg/kg dose of AZD-8529 did not reduce the number of rewards earned compared to the baseline and rebaseline sessions [Session: *F* < 1]. By contrast, the 10 mg/kg dose reduced the number of rewards earned during the drug treatment session [Session: *F*_(2,60)_ = 4.65, *p* =.013, η^2^ = 0.13] as compared to the rebaselining sessions (*p* =.012), though not the baselining sessions (*p* =.059).

Thus, of the doses of AZD-8529 tested, only the 3 mg/kg dose selectively reduced checking behaviour while leaving other measures of task performance intact.

### The mGluR2/3 agonist LY404039 dose-dependently reduced checking behaviour

A subset of the rats that had been tested with AZD-8529 were subsequently tested with different doses of the mGluR2/3 agonist LY404039 after a washout period. As before, checking behaviour was highly variable across individuals, so the data were square root transformed prior to analysis.

**Fig. 4:**
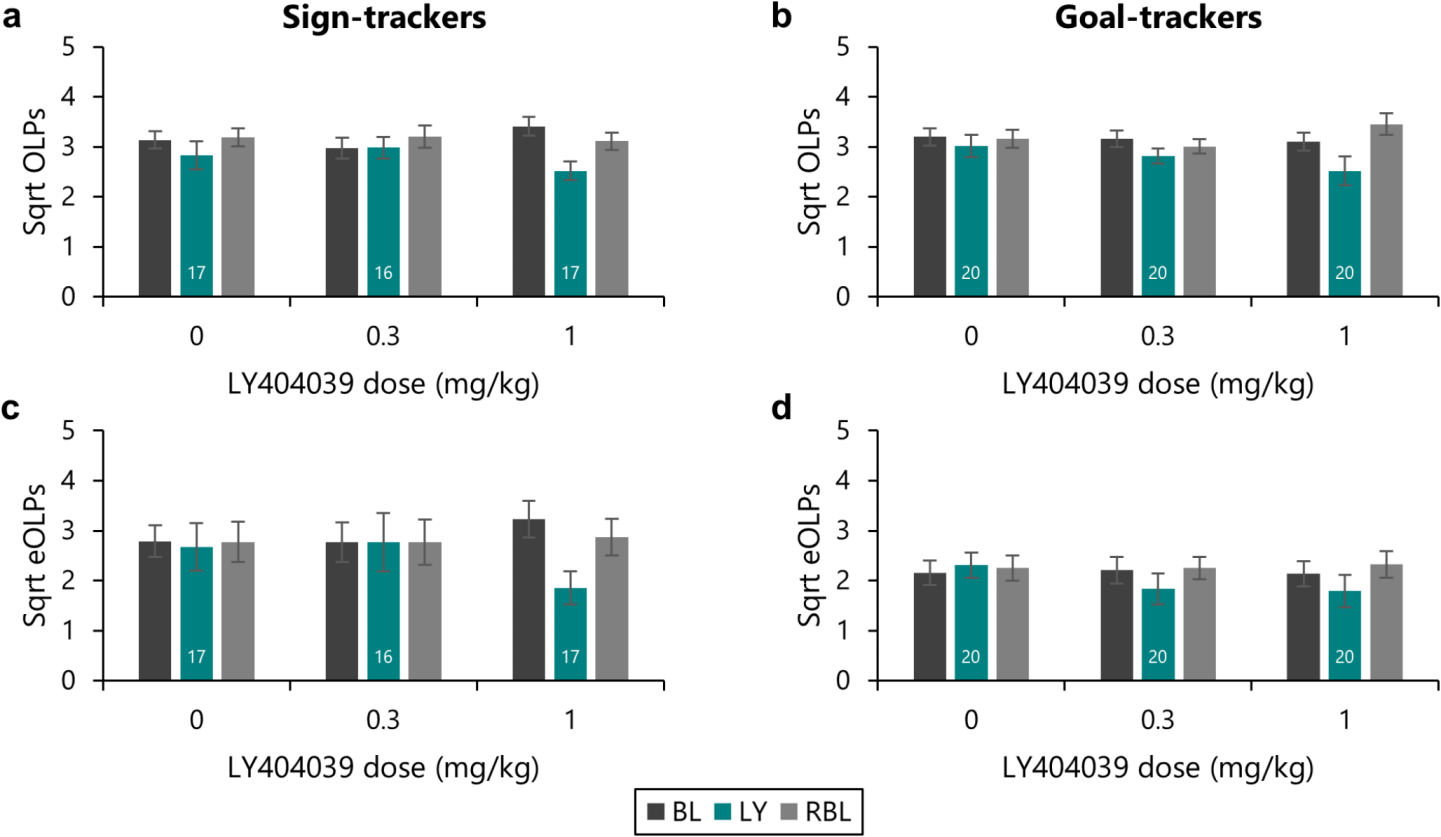
The mGluR2/3 agonist LY404039 dose-dependently reduced functional and dysfunctional checking. A subset of the rats tested with AZD-8529 were subsequently tested with LY404039 in a Latin square design. All rats received treatment with vehicle (0 mg/kg), and a subset of the different LY404039 doses. The number of rats receiving each dose is represented by numbers at the base of the bar for the drug dosing day; the same rats are represented in the ‘baseline’ and ‘rebaseline’ bars for each dose. In rats classified as sign-trackers, the 1 mg/kg dose of LY404039 reduced **(a)** functional Observing Lever Presses (OLPs) and **(c)** dysfunctional extra Observing Lever Presses (eOLPs). A similar effect was observed in goal-trackers, for both **(b)** functional OLPs and **(d)** dysfunctional eOLPs. BL, baseline sessions; LY: treatment with LY404039 treatment; RBL, rebaseline sessions. Data are shown as means ± s.e.m.

As expected, when administered with the double distilled water vehicle, levels of functional checking remained consistent across the baseline, testing and rebaselining sessions [Figure 4a, b; Session: *F*_(1.71,59.9)_ = 2.81, *p* =.076]. Functional checking was similarly unaffected by the 0.3 mg/kg dose of LY404039 [Session: *F*_(2,68)_ = 2.31, *p* =.11]. However, functional checking was reduced by the 1 mg/kg dose [Session: *F*_(1.66,58.0)_ = 18.9, *p* <.001, η^2^ = 0.35; pairwise comparisons showed that checking in the drug testing session was lower than the baseline and rebaseline sessions (all *p*’s < 0.001), which did not differ from each other (*p* =.99)].

A similar pattern was observed with dysfunctional checking (Fig. 4c, d). As expected, the vehicle injection produced no change in dysfunctional checking [Session: *F* < 1], and the 0.3 mg/kg dose also left dysfunctional checking intact [Session: *F* < 1]. However, as for functional checking, the 1 mg/kg dose reduced dysfunctional checking relative to the baseline and rebaseline sessions [Session: *F*_(2,70)_ = 14.1, *p* <.001, η^2^ = 0.29; pairwise comparisons showed reduced dysfunctional checking in the drug testing session relative to the baseline and rebaseline sessions (*p*’s < 0.001), which did not differ from each other (*p* =.95)].

Thus, the mGluR2/3 agonist LY404039 reduced both functional and dysfunctional checking behaviour at the 1 mg/kg dose.

### The 1 mg/kg dose of LY404039, which reduced checking, left generalised measures of task performance intact

#### Discrimination between the active and inactive levers, and overall rates of lever pressing

**Fig. 5:**
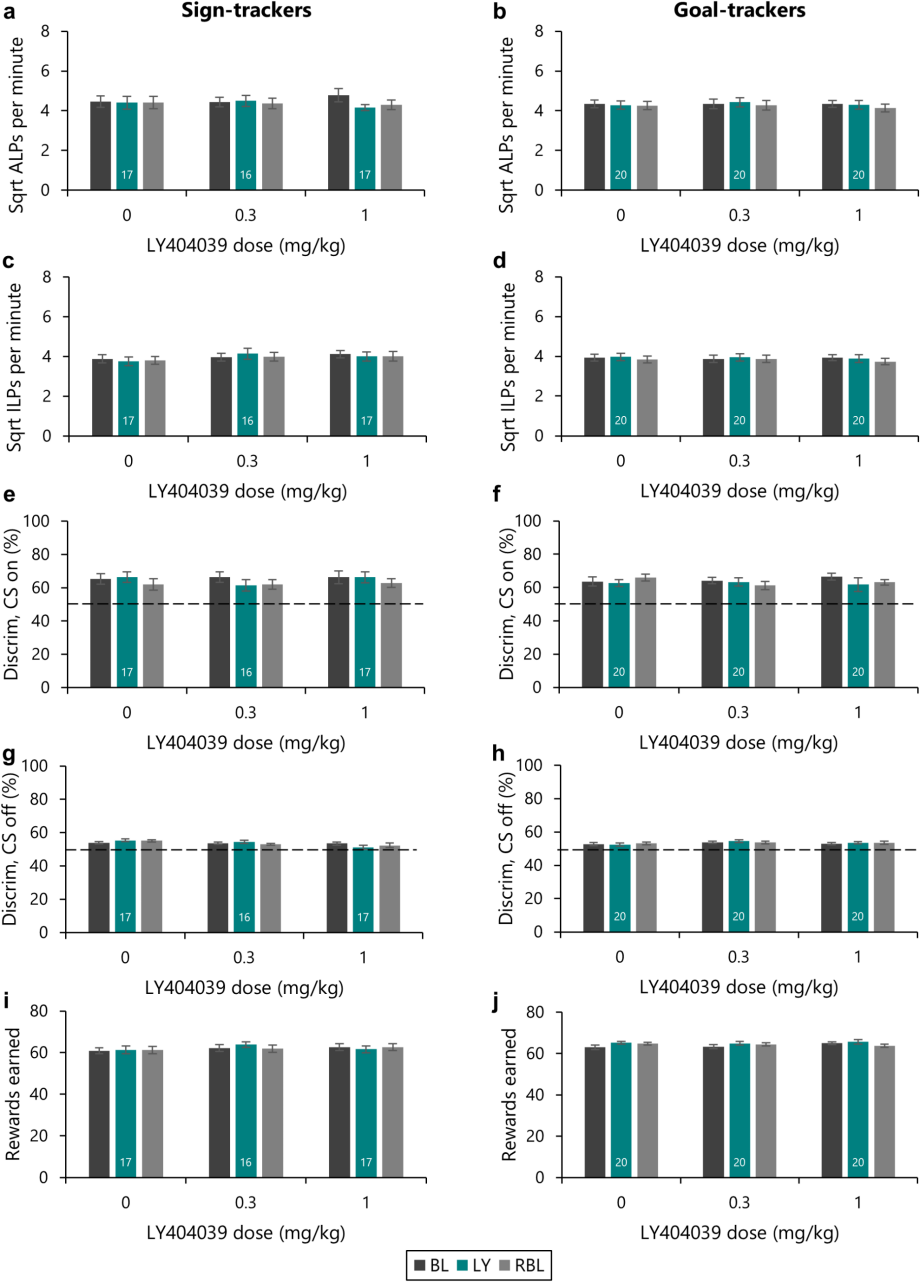
The reductions in checking produced by LY404039 were selective at the 1 mg/kg dose. The effects of LY404039 on generalised measures of task performance were assessed. For both sign-trackers **(a**,** c**,** e**,** g**,** i)** and goal-trackers **(b**,** d**,** f**,** h**,** j)** the 1 mg/kg dose of LY404039, which reduced checking, did not affect rates of **(a**,** b)** active lever pressing, or **(b**,** d)** inactive lever pressing, or **(e**,** f)** discrimination between the two levers with the cue light on. For both sign-trackers and goal-trackers, **(g**,** h)** discrimination with the cue light off remained at chance. **(i**,** j)** The number of rewards earned on task was not affected by the LY404039 doses tested. Group sizes are shown by the numbers at the base of each bar for the drug dosing day; the same rats are represented in the ‘baseline’ and ‘rebaseline’ bars for each dose. The dotted lines in (e), (f), (g) and (h) represent responding at chance (50%). Data are shown as means ± s.e.m

As would be expected, the vehicle treatment had no impact on the preference of rats for the active lever, or overall rates of lever pressing on the Observing Response Task (Fig. 5a-d). Rats pressed the active lever more [Lever: *F*_(1,35)_ = 66.9, *p* <.001, η^2^ = 0.66] and there were no differences in their rates of lever pressing across the baseline, test and rebaseline sessions [Session: *F* < 1; Lever x Session: *F* < 1]. This was also the case for the 0.3 mg/kg dose [Lever: *F*_(1,34)_ = 100.6, *p* <.001, η^2^ = 0.75; Session: *F*_(1.59,54.1)_ = 2.50, *p* =.10; Lever x Session: *F* < 1], which had also had no impact on checking behaviour.

The 1 mg/kg dose of LY404039, which reduced checking behaviour, did not affect the preference of rats for the active lever, or rates of lever pressing in the drug testing session. Rats continued to prefer the active over the inactive lever [Lever: *F*_(1,35)_ = 76.0, *p* <.001; η^2^ = 0.69]. Although the rates of lever pressing differed across the baseline, drug testing and rebaselining sessions [Session: *F*_(1.56,54.5)_ = 4.57, *p* =.022, η^2^ = 0.12; Lever x Session: *F*_(2,70)_ = 3.63, *p* =.032, η^2^ = 0.09; Lever x Session x Phenotype: *F*_(2,70)_ = 3.18, *p* =.048, η^2^ = 0.08], pairwise comparisons revealed no difference in the rate of lever pressing between the baseline and drug testing sessions [*p* =.18], but rather that rates of lever pressing were reduced in the rebaselining sessions, as compared to the pre-drug baseline [*p* <.001]. This reduction in the rate of lever pressing during the rebaseline sessions was specific to the active lever, on which responding was lower in the rebaseline sessions compared to both the baseline [*p* =.043] and the drug testing [*p* <.001] sessions, while rates of inactive lever pressing were unaffected [all *p*’s > 0.20]. These differences were driven by changes in the rate of lever pressing by the sign-tracking rats, which showed lower responding on the active lever in the rebaseline session compared to the baseline and drug testing sessions [*p*’s < 0.01] while the goal-tracking rats showed no change [all *p*’s > 0.11].

#### Discrimination with the cue light on and off

A similar pattern of results was observed for the capacity of rats to use the cue light to guide their responding towards the currently active lever (Fig. 5e-h). Under vehicle treatment, this remained unaffected [Cue: *F*_(1,35)_ = 43.5, *p* <.001, η^2^ = 0.55; Session: *F* < 1; Cue x Session: *F* < 1], as was also the case with the 0.3 mg/kg dose of LY404039 [Cue: *F*_(1,34)_ = 29.8, *p* <.001, η^2^ = 0.47; Session: *F*_(2,68)_ = 2.33, *p* =.11; Cue x Session: *F*_(2,68)_ = 2.18, *p* =.12]. Discrimination also remained intact under the 1 mg/kg dose of LY404039 [Cue: *F*_(1,35)_ = 46.0, *p* <.001, η^2^ = 0.57; Session: *F*_(1.35,47.3)_ = 1.05, *p* =.33; Cue x Session: *F* < 1].

#### Rewards earned

The number of rewards earned on task was also affected dose-dependently by LY404039 (Fig. 5i, j). As expected, the number of rewards earned was similar under vehicle as compared to the baseline and rebaseline sessions [Session: *F*_(1.38,48.2)_ = 1.23, *p* =.29], with goal-trackers earning more rewards overall [Phenotype: *F*_(1,35)_ = 4.44, *p* =.042, η^2^ = 0.11; Session x Phenotype: *F* < 1]. The 0.3 mg/kg dose of LY404039 led to a slight increase in the number of rewards earned relative to the baseline sessions [Session: *F*_(2,68)_ = 3.88, *p* =.025, η^2^ = 0.10; pairwise comparisons revealed that the number of rewards earned was higher in the drug test session than the baseline (*p* =.039) though not the rebaseline (*p* =.22) sessions]. The 1 mg/kg dose, which reduced checking, did not affect the number of rewards earned [Session: *F* < 1].

Thus, the 1 mg/kg dose of LY404039 reduced both functional and dysfunctional checking without impairing generalised task performance.

## Discussion

As observed previously (Eagle et al. 2020; Vousden et al. 2020), the Observing Response Task produced excessive and unnecessary checking behaviour, particularly in sign-tracking rats. Both functional and dysfunctional checking behaviour was dose-dependently reduced by systemic administration of both the mGluR2 positive allosteric modulator AZD-8529, and the mGluR2/3 agonist LY404039 (pomaglumetad). For both drugs, it was possible to titrate a dose that selectively reduced checking behaviour, without impairing other measures of ongoing task performance such as the rates of lever pressing for sucrose rewards, capacity to use the cue light to guide behaviour, or the number of rewards earned.

These data support the value of treatments modulating glutamatergic signalling for the amelioration of OCD symptoms. The similarity of effect between the mGluR2 positive allosteric modulator and the mGluR2/3 agonist suggests that the reduction in checking behaviour is mediated by action at the mGluR2 subtype of receptor, rather than mGluR3. Despite the similarity in their genetic sequences, intracellular signalling effects via the G_i_-subtype of G-protein, and presynaptic localisation (Ohishi et al. 1993a, b; Schoepp 2001; Tanabe et al. 1992), mGluR2 and mGluR3 are differentially expressed in other synaptic sites. Specifically, mGluR3 is expressed both on astrocytes, where it facilitates glial glutamate reuptake via regulation of excitatory amino acid transporters (Aronica et al. 2003) and postsynaptically (Jin et al. 2018; Ohishi et al. 1993b). Thus, although agonism at mGluR3 might enhance the effectiveness of LY404039 through enhanced glutamate reuptake, the similarity in effect of the LY404039 with the mGluR2-selective positive allosteric modulator AZD-8529 suggests that the reduction in checking behaviour is primarily mediated via mGluR2s.

Importantly, relatively selective effects on checking were obtained at doses lower than have been shown effective in other behavioural tests, such as nicotine-seeking (Li et al. 2016), amphetamine-induced hyperlocomotion, conditioned avoidance or marble burying (Rorick-Kehn et al. 2007). Marble burying has been used as a behavioural assay for drugs suitable for treatment of OCD (Angoa-Pérez et al. 2013); however, decremental effects of LY404039 were found only at 3 mg/kg (hence discontinuation of testing of this dose), in contrast to the selective effects shown for 1 mg/kg in the ORT. Thus, the ORT may be a more sensitive test for this purpose, especially as it is based on a similar decision-making task already used for OCD patients (Morein-Zamir et al. 2018), whereas this is not the case for marble-burying.

As both compounds were administered systemically in the present study, it is not possible to determine the specific neural sites at which mGluR2 modulation was exerting its effects. Both mGluR2 and mGluR3 are widely expressed in the brain, with both receptors being highly expressed in key neural sites implicated in OCD, including the striatum, nucleus accumbens and cingulate cortex (Tamaru et al. 2001), although with differential expression in the subthalamic nucleus (Tamaru et al. 2001; Testa et al. 1994). It is possible to speculate that an important target for the mGluR drugs might be the anterior cingulate cortex, which has been shown by magnetic resonance spectroscopy to exhibit higher glutamate: GABA ratios in human OCD patients (Biria et al. 2024), especially as a human fMRI study has shown that AZD-8529 enhances anterior cingulate activation in patients with schizophrenia performing an *n*-back working memory task (Wolf et al. 2022).

Considering the reliable relationship between sign-tracking and excessive dysfunctional checking (Eagle et al. 2020; Pickenhan and Milton 2024; Vousden et al. 2020), and the reliance of autoshaping on a nucleus accumbens-anterior cingulate circuit (Parkinson et al. 2000), it is tempting to speculate that the effects of AZD-8529 and LY404039 on checking behaviour might be through a reduction of presynaptic glutamate release within the anterior cingulate cortex. However, it remains to be demonstrated whether the drugs targeting mGluR2s could produce the same effect if infused locally into the anterior cingulate cortex, or whether dysfunctional checking is associated with increased glutamate: GABA ratios within the anterior cingulate. These would be interesting avenues for future research. Furthermore, we acknowledge the limitation of using the same manipulandum during autoshaping and as the checking lever on the ORT, which makes it unclear whether sign-trackers check more due to a greater reliance on habitual learning, or because the lever has acquired excessive incentive salience in these animals. The use of an alternative manipulandum during autoshaping would help to overcome this limitation. We note, however, that while the effects of the mGluR2-targeting drugs were clearer in the sign-trackers (due to higher checking baselines), the drugs tested were also effective at reducing checking (albeit from a lower initial level) in goal-trackers.

Both AZD-8529 and LY404039 have been shown to be safe for use in humans, albeit that LY404039 has been administered in an altered form with better oral bioavailability (LY2140023; Patil et al. 2007). AZD-8529 has been tested in human clinical trials assessing its impact on positive and negative symptoms in schizophrenia (Litman et al. 2016) and nicotine use disorder (see Lassi et al. 2016, for review). Though AZD-8529 did not improve clinical outcomes in either of those trials, the drug did affect fMRI measures predictive of symptoms (Wolf et al. 2022) and adverse effects were mild (e.g. headaches) although it is of note that some patients reported gastrointestinal upset. This is consistent with the reduced food self-administration found in rats given chronic high (58 mg/kg) doses of AZD-8529 (Li et al. 2016) and our finding that a 30 mg/kg dose of AZD-8529 impaired gastrointestinal motility in rats. However, the dose of AZD-8529 required to reduce checking behaviour (3 mg/kg) was markedly lower than the one that caused adverse effects in our animals, suggesting a large safety margin. Similarly, the LY404039-derivative LY2140023 has been trialled for the treatment of schizophrenia (Patil et al. 2007) and was reported to have minimal side effects, though some gastrointestinal adverse effects (e.g. vomiting, dyspepsia) were noted in patients receiving LY2140023 (Adams et al. 2013). Thus, both AZD-8529 and LY404039 appear likely safe to use in humans at the doses required to reduce checking behaviour in rats, though more research would be required to determine the safety of chronic dosing, which would likely be required due to the acute reductions in checking observed in the drug-treatment session in this study.

Here we demonstrated that two drugs targeting mGluR2s can dose-dependently modulate checking behaviour in rats performing a behavioural task with high translational relevance to OCD (Pickenhan and Milton 2024). The reduction in checking was selective, not affecting other measures of task performance. The results of the current study suggest that mGluR2s may provide a novel therapeutic target to reduce the overly high glutamatergic signalling observed in patients with OCD.

## Electronic supplementary material

Below is the link to the electronic supplementary material.

Supplementary Material 1

Supplementary Material 2

## Data Availability

Data are available at 10.17863/CAM.111643.
