# Supplementary figures and images for: Agonism at mGluR2 receptors reduces dysfunctional checking on a rodent analogue of compulsive-like checking in obsessive compulsive disorder
Source: Psychopharmacology (Berl). 2025 Apr 3;242(8):1893–907. doi: 10.1007/s00213-025-06774-2 (PMC12296766; doi:10.1007/s00213-025-06774-2)

## Slide 1
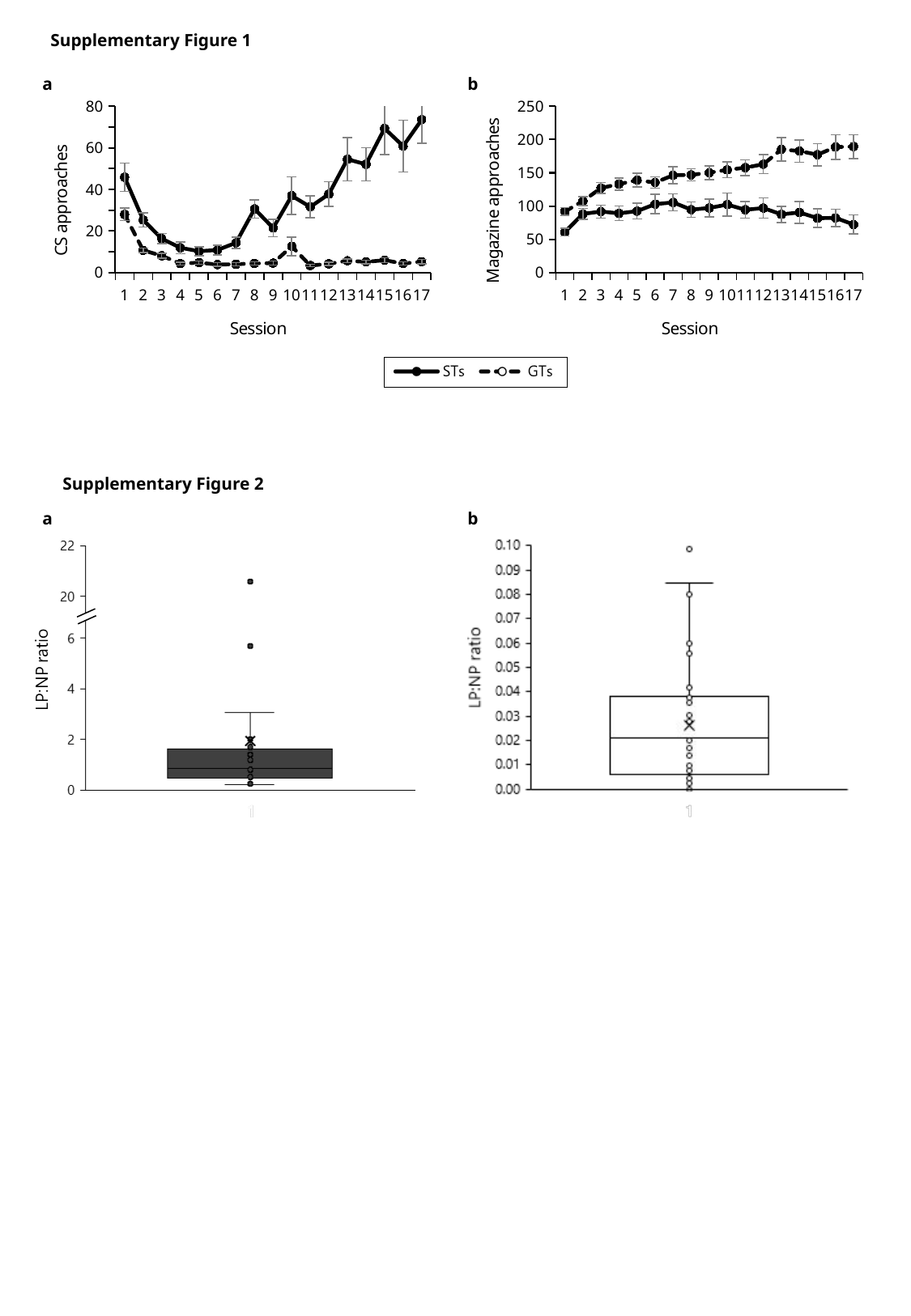

Supplementary Figure 1
b
a
### Chart
| Category | | |
|---|---|---|
### Chart
| Category | | |
|---|---|---|
Supplementary Figure 2
b
a

Supplement: Supplementary file 1 — Supplementary Material 1 [file 213_2025_6774_MOESM1_ESM.pptx]
