# Supplementary material for: Agonism at mGluR2 receptors reduces dysfunctional checking on a rodent analogue of compulsive-like checking in obsessive compulsive disorder
Source: Psychopharmacology (Berl). 2025 Apr 3;242(8):1893–907. doi: 10.1007/s00213-025-06774-2 (PMC12296766; doi:10.1007/s00213-025-06774-2)
Supplement: Supplementary file 2 — Supplementary Material 2 [file 213_2025_6774_MOESM2_ESM.docx]

**SUPPLEMENTARY FIGURE LEGENDS**

**Supplementary Figure 1.** Sign-trackers and goal-trackers showed differences in both **(a)** CS approaches and **(b)** magazine approaches during the 17 sessions of pavlovian autoshaping. Sign-trackers showed higher levels of CS approach than goal-trackers [Phenotype: *F*_(1,67)_ = 64.9, *p* < .001, η^2^ = 0.49]. These differences were apparent across training, and increased over time [Session: *F*_(4.4,293)_ = 22.1, *p* < .001, η^2^ = 0.25; Session x Phenotype: *F*_(4.4, 293)_ = 19.2, *p* < .001, η^2^ = 0.22]. By contrast, goal-trackers showed greater levels of magazine approach [Phenotype: *F*_(1,67)_ = 15.3, *p* < .001, η^2^ = 0.19], with these differences being clear at the start of training and increasing over time [Session: *F*_(3.9,261)_ = 6.53, *p* < .001, η^2^ = 0.09; Session x Phenotype: *F*_(3.9,261)_ = 6.15, *p* < .001, η^2^ = 0.084].

**Supplementary Figure 2.** Sign-trackers and goal-trackers were classified on the ratio of lever pressing (LP) and magazine approaches (nosepokes; NP), with the average of the final two days of autoshaping being used to generate an LP:NP ratio. **(a)** Boxplot of individual sign-tracker rat averages. For ease of interpretation, the y-axis is broken. **(b)** Boxplot of individual goal-tracker rat averages.
